# Increased risk of ventricular tachycardia and cardiovascular death in patients with myocarditis during the long-term follow-up: A national representative cohort from the National Health Insurance Research Database

**DOI:** 10.1097/MD.0000000000006633

**Published:** 2017-05-05

**Authors:** Abigail Louise D. Te, Tao-Cheng Wu, Yenn-Jiang Lin, Yun-Yu Chen, Fa-Po Chung, Shih-Lin Chang, Li-Wei Lo, Yu-Feng Hu, Ta-Chuan Tuan, Tze-Fan Chao, Jo-Nan Liao, Kuo-Liong Chien, Chin-Yu Lin, Yao-Ting Chang, Shih-Ann Chen

**Affiliations:** a Division of Cardiology, Department of Medicine, Taipei Veterans General Hospital; b School of Medicine, Institute of Clinical Medicine and Cardiovascular Research Center, National Yang-Ming University; c Institute of Epidemiology and Preventive Medicine College of Public Health, National Taiwan University, Taipei, Taiwan.

**Keywords:** mortality, myocarditis, ventricular tachycardia

## Abstract

The incidence of acute myocarditis complicated with ventricular tachycardia (VT) is unknown. This study aimed to investigate the association between myocarditis and the incidence of VT and mortality. We also aimed to determine the independent predictors that increased the VT risk in those patients. From 2000 to 2004, 13,250 patients with a history of myocarditis were identified from the Taiwan National Health Insurance Research Database. The same number of individuals without heart disease with a matched sex and underlying diseases were selected as the control group. The long-term risks of life-threatening ventricular arrhythmias and mortality in patients with a history of myocarditis were investigated by an adjusted Cox proportional hazards regression. After a mean follow-up of 10.4 ± 2.94 years (interquartile range: 12, 10.19–12), the myocarditis patients showed a higher incidence of new onset VT events compared with healthy controls (5.4% [519 per 100,000 person-year] in the myocarditis group vs, 0.47% [43 per 100,000 person-year] in the healthy controls; adjusted hazard ratio [HR]: 16.1, 95% confidence interval [CI]: 12.4–20.9; *P* < .001). A higher incidence of cardiovascular death was noted in the myocarditis group than healthy controls (6.52% vs 3.18%; HR: 2.42, 95% CI: 2.14–2.73; *P* < .001) after adjusting for the multivariate confounders including sex, age, underlying comorbidities, and medications. The results of this study suggested that there was higher incidence of life-threatening VT and mortality during the very long-term follow-up in patients with a history of myocarditis. Future work should focus on an in-depth risk stratification of VT in myocarditis patients.

## 1 Introduction

Myocarditis is a common cardiac disease that is identified in up to 9% of routine postmortem examinations.^[1]^ It can present with heterogeneous clinical manifestations from nonspecific symptoms to a rapidly deteriorating cardiac function and life-threatening arrhythmias.^[2]^ In patients with a benign course of the disease, the cardiac function may completely recover or progress to chronic dilated cardiomyopathy.^[3]^ Long-term follow-up studies^[4–6]^ of patients with a history of acute myocarditis often have a 5- or 6-year follow-up period, and those studies looked into the association of the development of chronic dilated cardiomyopathy and the risk of sudden cardiac death. Other long-term studies were limited to a certain etiology of the myocarditis and identification of the long-term predictors of the mortality using several parameters from diagnostic studies.^[4,7]^ However, the incidence of new-onset ventricular tachycardia (VT) during a 10-year follow-up period especially in patients who recovered from acute myocarditis without any evident clinical cardiovascular (CV) sequelae is presently unknown. In addition, no study has been able to show the overall mortality risk in these patients during the very long-term follow-up. The purpose of this study was to investigate the association between acute myocarditis and the incidence of VT and mortality during the long-term follow-up period from the National Health Insurance Research Database (NHIRD) of Taiwan.

## 2 Methods

### 2.1 Study design and participants

This study was a population-based retrospective cohort study. From January 1, 2000 to December 31, 2004, a total of 13,250 patients aged 18 years and older, who were diagnosed with myocarditis, were identified from the NHIRD according to the International Classification of Diseases, 9th Revision—Clinical Modification (ICD9-CM) codes (422). The diagnosis of myocarditis must be recorded twice in the outpatient records or at least once in the inpatient records. On the same index date, the same number of health controls without prior structural heart disease, matched by the sex, history of hypertension (HTN), diabetes mellitus (DM), chronic obstructive pulmonary disease (COPD), chronic kidney disease (CKD), hyperlipidemia, and thyroid disease, were selected to be the control group for each study patient. Patients who were diagnosed with ischemic heart disease, heart failure, valvular heart disease, congenital heart disease, overt cardiogenic shock requiring vasopressors at the initial presentation had a history of ventricular arrhythmia or previous implantable cardioverter defibrillator (ICD) implantation, and those with systemic inflammatory diseases known to be associated with myocardial involvement were excluded from the study. The comorbid conditions of each individual were retrieved from the medical claims database based on the ICD9-CM codes.

### 2.2 Database

This study used the Taiwan NHIRD to determine the risk of ventricular tachycardia (VT) and CV death in patients with myocarditis during the long-term follow-up (from 2000 to 2011). The Taiwan Collaboration Center of Health Information Application, Ministry of Health and Welfare, provided the entire datasets used in this study. The Taiwan's National Health Insurance (NHI) program enrolled 23 million people, which covered 99% of the country's population and contained data on utilization of all NHI resources, including outpatient visits, hospital care, prescribed medications, and the National Death Registry. It contained all the medical claims data for 1000,000 beneficiaries, who were randomly sampled from the 25.68 million enrollees under the NHI program. These random samples have been confirmed by the NHRI to be representative of the Taiwanese population. The protocol was reviewed and approved by the Research Ethics Committee of National Taiwan University Hospital (NTUH-Rec Number: 201305044W [Institutional Review Board reference]). In addition, we obtained permission for the rights from the National Research Institute for the Department of Health and the Health Promotion Administration, Ministry of Health and Welfare.

### 2.3 Study endpoints during the follow-up

The follow-up period ended when the subjects developed new onset VT, death, or lived beyond December 31, 2011. The primary endpoints were the time to the development of new-onset VT and the time to CV death/all-cause mortality during the follow-up. Death was confirmed by referencing the Taiwan's National Death Registry. The time to implantation of an ICD was investigated as a secondary endpoint.

### 2.4 Statistical analysis

The normally distributed continuous variables were presented as the mean values and standard deviation and were compared using a Student *t* test. The non-normally distributed variables were compared using the Mann–Whitney *U* test. Frequencies were compared using the chi-square test. The incidence rates of CV events were calculated as the number of cases per 100,000 person-years of follow-up. In order to minimize the impact of the confounding factors on the clinical characteristics, we employed the propensity analysis and matching technique. We matched the pairs one-to-one with identical propensity scores with a 0.01 caliper width for the sex, HTN, DM, CKD, COPD, hyperlipidemia, and thyroid disease.

The event-free survival curve was plotted using the Kaplan–Meier method with the statistical significance examined by the log-rank test. The Cox proportional-hazards regression was used to compare the hazard ratios (HR) with 95% confidence intervals (CIs) for the outcomes. Potential confounders were adjusted via 3 models. Model 1: age and sex; Model 2: Model 1 plus HTN, DM, COPD, CKD, hyperlipidemia, and thyroid disease; Model 3: Model 2 plus medications, including angiotensin-converting enzyme inhibitors (ACEis), angiotensin receptor II blockers (ARBs), and beta-blockers (BBs). The level of statistical significance was set at a 2-tailed alpha level <0.05. The analyses were performed with SAS version 9.3 software (SAS Institute, Cary, NC).

## 3 Results

### 3.1 Patient characteristics

A total of 13,250 patients with a history of myocarditis and 13,250 healthy controls without a prior history of structural heart disease were identified as the study population with a mean follow-up of 10.4 ± 2.94 years (interquartile range: 12, 10.19–12). The characteristics of the study population are shown in Table 1. The baseline characteristics of the two populations did not significantly differ except for the age wherein the patients with myocarditis were younger than the healthy cohort (myocarditis group, 43 ± 27 years old vs healthy cohort, 44 ± 26 years old, *P* < .01). In addition, the use of antihypertensive medications, particularly ACEis, ARBs, and BBs, were significantly more frequent in the myocarditis group than in the healthy cohorts (ACEi: 15.11% patients with myocarditis vs 0.01% healthy cohorts, *P* < .001; ARB: 3.97% vs 0.02%, *P* < .001; BB: 16.96% vs 0.04%, *P* < .001).

**Table 1:**
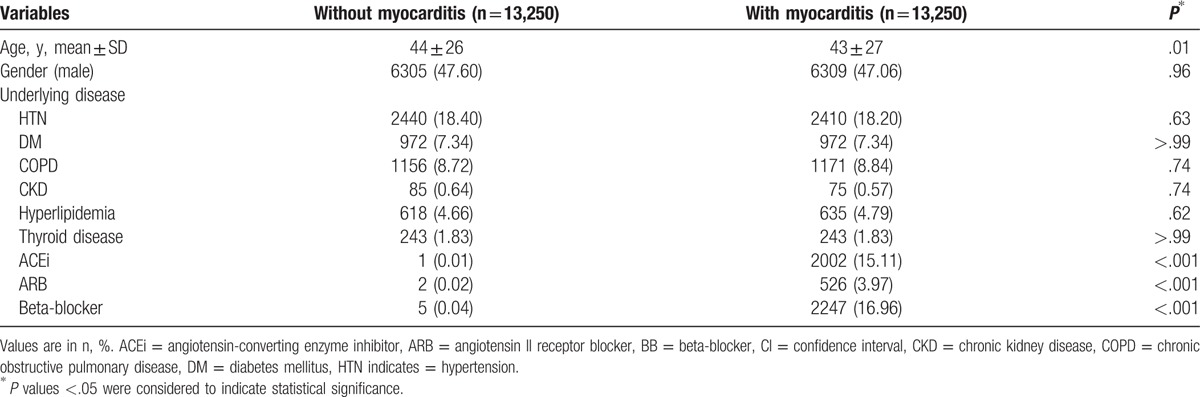
Characteristics of patients with and without myocarditis.

### 3.2 Incidence of events

The patients with myocarditis had a higher incidence of new onset VT events than the healthy controls (5.40% [519 per 100,000 person-year] in myocarditis group vs 0.47% [43 per 100,000 person-year] in healthy controls; adjusted HR: 16.06, 95% CI: 12.37–20.86; *P* < .001). Higher incidences of CV death (6.52% vs 3.18%; adjusted HR: 2.42, 95% CI: 2.14–2.73; *P* < .001; Table 2) and all-cause mortality (24.5% vs 18.9%; adjusted HR: 1.41, 95% CI: 1.33–1.49; *P* < .001) were also noted in the myocarditis group than the healthy cohort. The incidence of ICD implantations was also significantly higher in the myocarditis group (0.13% vs 0.02%; adjusted HR: 12.07, 95% CI: 2.74–53.08, *P* < .001) than the healthy control population after adjusting for multivariate confounders including the sex, age, underlying comorbidities, and medications (Table 2).

**Table 2:**
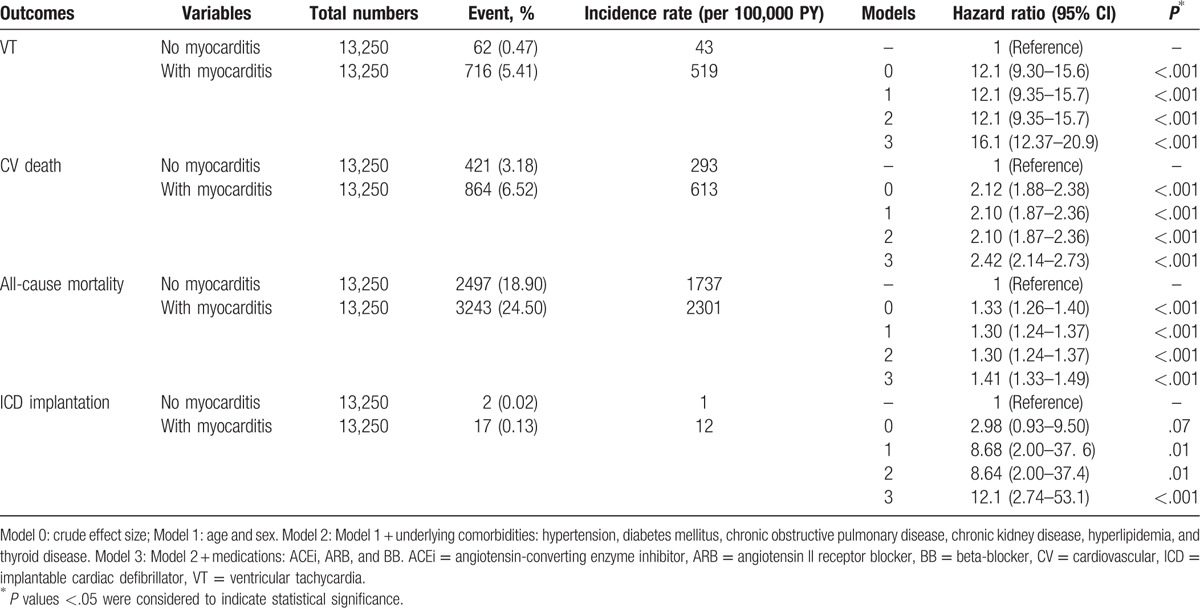
Incidence rate of primary and secondary outcomes in the patient population.

### 3.3 Event-free survival

**Figure 1:**
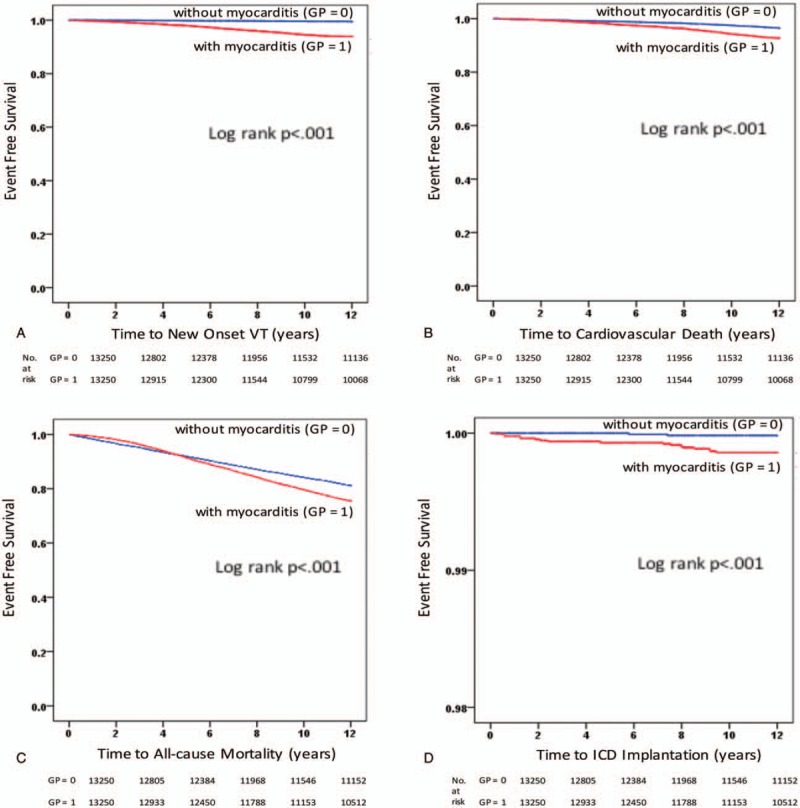
Kaplan–Meier event-free survival curves for (A) new-onset ventricular tachycardia events, (B) cardiovascular death, (C) all-cause mortality, and (D) implantable cardioverter defibrillator implantations comparing the patients with and without myocarditis. In the log-rank test, a *P* value of <.05 was considered as significant.

The Kaplan–Meier event-free survival curves for new-onset VT events, CV death, all-cause mortality, and ICD implantations comparing patients with and without myocarditis are shown in Fig. 1A–D. There were significant differences in the new-onset VT events, CV death, and all-cause mortality between the two groups (Fig. 1A–C) with the myocarditis groups showing increased new-onset VT events and decreased survival compared to the healthy controls. In addition, the overall risk for mortality (Fig. 1C) for the two groups was initially similar (from index event up to follow-up of 5 years). However, 5 years onward in the course of the follow-up, the Kaplan–Meier curve demonstrated a divergence in the survival curve between the myocarditis group and healthy controls, wherein the myocarditis group had a significantly increased overall mortality risk (log rank *P* < .001), whereas the healthy controls’ mortality risk remained the same. The risk for the ICD implantations was also significantly higher in the myocarditis group than in the healthy controls during long-term follow-up (log rank *P* < .001).

### 3.4 Predictors of the occurrence of ventricular arrhythmias

The characteristics of the patients with myocarditis who developed new-onset VT during the follow-up are shown in Table 3. Those who developed VT were mostly males (with VT, 55.30% vs without VT, 47.20%, *P* < .001), had DM (10.20% vs 7.17%, *P* = .01), CKD (2.51% vs 0.45%, *P* < .001), hyperlipidemia (14.30% vs 4.25%, *P* < .001), and a significantly lesser use of ACEis (4.05% vs 15.7%, *P* < .001), ARBs (2.23% vs 4.07%, *P* < .001), and BBs (4.89% vs 17.7%, *P* < .001). A multivariable Cox proportional hazard regression analysis revealed that the independent predictors that were significantly associated with new-onset VT events in patients with a history of myocarditis had an older age (adjusted HR: 1.003, 95% CI: 1.00–1.006, *P* = .04), male gender (adjusted HR: 1.33, 95% CI: 1.15–1.54, *P* < .001), presence of DM (adjusted HR: 1.40, 95% CI: 1.09–1.79, *P* = .01), CKD (adjusted HR: 2.91, 95% CI: 1.81–4.68, *P* < .001), hyperlipidemia (adjusted HR: 3.05, 95% CI: 2.46–3.78, *P* < .001), and lesser use of ACEis (adjusted HR: 0.25, 95% CI: 0.17–0.37, *P* < .001), ARBs (adjusted HR: 0.53, 95% CI: 0.32–0.88, *P* = .01), and BBs (adjusted HR: 0.26, 95% CI: 0.19–0.37, *P* < .001).

**Table 3:**
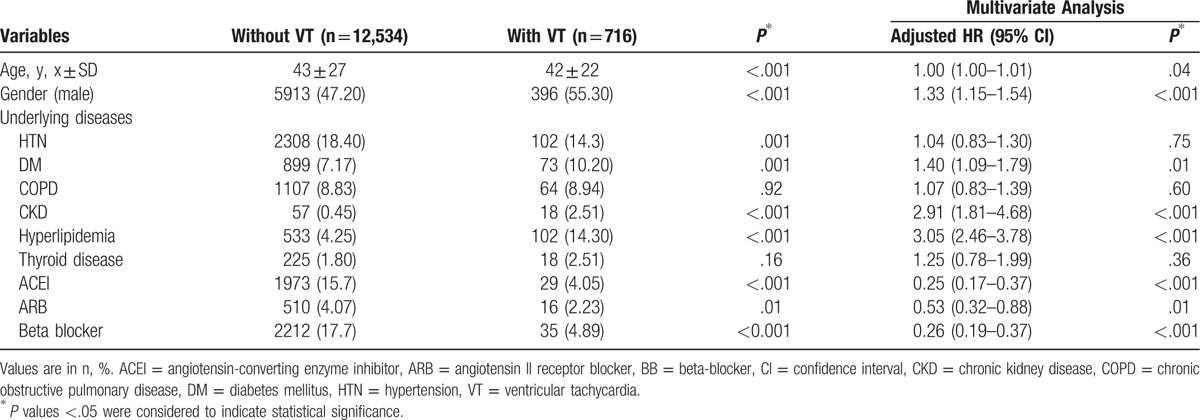
Characteristics of patients with myocarditis with and without ventricular tachycardia.

## 4 Discussion

### 4.1 Main findings

The main findings of the study are as follows. First, from the national database, this study suggested a higher incidence of life-threatening VT, mortality, and ICD implantations during the very long-term follow-up in patients with a history of acute myocarditis, after adjusting for the multivariate confounders. Second, the predictors of new-onset of VT were a younger age, male gender, presence of DM, CKD, hyperlipidemia, and a lesser use of ACEis, ARBs, and BBs. Third, the concomitant use of ACEis, ARBs, and BBs may provide a protective role from life-threatening VT during the long-term follow-up in patients with a previous history of myocarditis.

### 4.2 Compared with the previous findings

Myocarditis contributes to the global burden of CV disease primarily through sudden cardiac death and dilated cardiomyopathy. Accurate population-based estimates of the myocarditis incidence and prevalence are not directly available in any world region.^[8]^ The long-term follow-up studies in patients with acute myocarditis with preserved left ventricle systolic function mostly focused on identifying the predictors of the development of dilated cardiomyopathy,^[5]^ which is the most common long-term sequela.^[9]^

In this study, we found that patients with a history of acute myocarditis had a 12-fold increase in new-onset VT during the very long-term follow-up compared to the healthy population without structural heart disease. This study also showed that those with a history of myocarditis had a 2-fold and 1.33-fold increase in the CV mortality and all-cause mortality, respectively. The overall survival for both groups was initially similar as depicted in the Kaplan–Meier curve (Fig. 1C). However, during the course of the follow-up, particularly 5 years onward, the survival curve started to progressively decline in the myocarditis group with a significantly increased risk of overall mortality (25%) compared to the healthy cohort (19%). Previous studies have reported that acute myocarditis, though patients may initially present less ill,^[4]^ causes substantial mortality with a 21% 1-year mortality rate after the presentation of myocarditis.^[5,9]^ These mortalities were due to chronic dilated cardiomyopathy secondary to a chronic myocardial inflammatory process. So far, no study has been able to show the long-term overall mortality risk for patients who have recovered from acute myocarditis without any evident clinical CV sequelae. Our study showed that those groups of patients have a significantly decreased long-term survival. Whether or not occult persistent inflammation plays a role in contributing to the overall long-term mortality in those patients, this distinction cannot be made based on the clinical grounds alone; thus, further study is recommended. In addition, this study showed that these patients have a 3-fold increased risk of ICD implantations with a significantly greater increased mortality than in the healthy cohort. The patients who may have required primary prevention for ICD implantations may show a higher CV mortality. Based on the Taiwan NHI, ICD implantations are indicated only for secondary prevention and not for primary prevention of VT events. This may be one of the reasons to explain the high mortality rate in this study. This study showed that the VT may occur 5 to 10 years after the index event of myocarditis, and the secondary prevention strategy may put these patients at risk for VT events.

### 4.3 Potential mechanism of VTs

Although VT is an uncommon initial manifestation of myocarditis, it often develops during the long-term follow-up, but few studies have reported this.^[10]^ The mechanism of VT may be attributed to the chronic myocardial inflammatory process secondary to pathogenic autoimmunity that may continue even after myocardial recovery.^[11,12]^ The mechanism of arrhythmogenesis seems to be the formation of micro-reentry circuits, favored by myocyte injury and replacement fibrosis^[13]^ and triggered activity mainly due to the proarrhythmic effects of cytokines which may continue even in the absence of overt inflammation.^[12]^ The environment surrounding the myocytes could also influence the electrophysiological properties of the myocardium, leading to the phenomenon known as electrical remodeling.^[13]^ These changes may completely or partially reverse after the healing of the inflammatory process. However, the exact duration and extent of this process are unknown inasmuch as the natural history of VTs in myocarditis has not been adequately studied and documented.

### 4.4 Predictors of VTs

Furthermore, our study showed that an older age, male gender, presence of DM, CKD, hyperlipidemia, and lesser use of ACEis, ARBs, and BBs were identified as independent risk factors for developing new-onset VT in patients with a history of myocarditis in the long-term follow-up. In our study, 55% of patients with a history of myocarditis who had VT were men. Although the exact physiologic mechanism that triggers this phenomenon is not clear, it is likely that men have a greater propensity to ventricular arrhythmias than women.^[14]^ Numerous studies in the general population have pointed out that men experience a higher rate of ventricular arrhythmias and sudden death compared to women.^[14]^ The differences in the electrophysiologic properties related to sex hormones may, at least in part, explain the gender-specificity propensity to ventricular arrhythmias.^[15]^

DM,^[16]^ CKD,^[17]^ and hyperlipidemia^[18]^ may individually or altogether overlap to increase the myocardial vulnerability to ventricular arrhythmias. Increases in the proinflammatory cytokines, inflammatory mediators, and reactive oxygen species during the disease process have direct effects on the myocardium resulting in myocardial fibrosis. This provides an additional substrate for an increased electrical instability and disturbances in the repolarization.^[19]^ Contrary to our knowledge that HTN is a risk factor for sudden cardiac death caused by ventricular tachycardia and fibrillation, our study showed that the presence of HTN in these group of patients have no significant predictive impact on the long-term risk for VT events. However, the intake of antihypertensive medications prescribed for HTN in these patients, particularly, ACEis, ARBs, and BBs may have provided protection from a long-term risk for VT events. Studies have shown that the remodeling of the ventricle that often occurs both in acute (i.e., myocarditis) and chronic (i.e., HTN and cardiomyopathy) clinical conditions^[19]^ may continue for months and years after the initial insult, regardless of the etiology. This remodeling can result in an increase in the susceptibility of developing ventricular arrhythmias. Previous studies have demonstrated the role of a pharmacologic treatment, particularly with ACEis, ARBs, aldosterone antagonists, and BBs, in reversing or slowing down the maladaptive ventricular remodeling,^[20]^ as well as their role in the prevention and treatment of ventricular arrhythmias. This may explain the protective role of these medications in our study patients.

The combination of a triggering event and a susceptible myocardium has been evolving as a fundamental electrophysiological concept for the mechanism of the initiation of a potentially lethal arrhythmia. However, much is still to be elucidated from the interaction of the long-term risk of ventricular tachycardia in myocarditis and these independent risk factors.

### 4.5 Clinical Implications

The natural history of myocarditis is as varied as its clinical presentation. The results of this study may have some important clinical implications. Our findings suggest that several years after admission for myocarditis, increasing events of new-onset VT and mortality could be observed in these patients. This implies that closely monitored outpatient follow-up visits may be necessary in order to provide timely management strategies to prevent the development or control of DM, CKD, and hyperlipidemia, especially among younger males. Furthermore, this study also showed the protective role of ACEis, ARBs, and BBs in preventing future VT events in those patients. Thus, for patient who has a history of myocarditis, these medications may be prescribed to reverse or slow down maladaptive ventricular remodeling in these patients.^[20]^

### 4.6 Study limitations

The present study showed the long-term survival difference between patients with a history of myocarditis and the healthy population without structural heart disease. Nonetheless, there were several limitations to our study. First, the diagnosis of myocarditis was based on the ICD-9 codes and was not further confirmed with endomyocardial biopsy results. Therefore, the diagnostic accuracy of myocarditis cannot be fully established. Second, our data did not include all the various therapies that patients may have received over the 10-year follow-up period. We could only account for the use of ACEis, ARBs, and BBs in our study population. Lastly, although we adjusted for confounders using the Cox proportional-hazard regression analysis, the retrospective nature of this study limits some of the conclusions that we can derive from the data. Future studies are recommended to show the causality of the independent predictors for new-onset VT during the long-term follow-up in patients with a history of acute myocarditis.

## 5 Conclusion

To the best of our knowledge, the present study represents the longest follow-up period in patients with a history of myocarditis. The results of this study suggest a higher incidence of life-threatening VT and mortality during a very long-term follow-up in patients with a history of acute myocarditis. It also suggests that ACEis, ARBs, and BBs may play a protective role in preventing future VT events in these patients. Therefore, future work should focus on an in-depth risk stratification of VT in myocarditis patients.
